# Patients’ perceptions of bariatric surgery in the Deep South: the impact of health literacy

**DOI:** 10.1007/s00464-025-11916-w

**Published:** 2025-07-08

**Authors:** Alfonsus Adrian Hadikusumo Harsono, Gina Kim, Nathan C. English, Gurudatta A. Naik, Dinakar Sai Velagala, Kristen Wong, Richard D. Stahl, Jayleen M. Grams, Daniel I. Chu, Margaux N. Mustian

**Affiliations:** 1 Division of Gastrointestinal Surgery, Department of Surgery, University of Alabama at Birmingham, 1808 7Th Ave S, BDB 564, Birmingham, AL 35294-0016 USA; 2 Department of Surgery, University of Cape Town, Cape Town, South Africa

**Keywords:** Bariatric surgery, Health literacy, Deep South

## Abstract

**Background:**

Bariatric surgery utilization is low in the Deep South despite the high prevalence of obesity in the region. The impact of health literacy (HL) on access to bariatric surgery is unclear. We aim to assess the relationship between HL and patients’ perspectives on healthcare, including bariatric surgical care in the Deep South.

**Methods:**

A multi-institutional study was conducted in surgery clinics at three institutions in Alabama. Participants were recruited to complete a survey based on the NIH PhenX tool kit, assessing socioeconomic determinants of health, as well as perception of safety, efficacy, and accessibility of bariatric surgery. HL was measured based on BRIEF Health Literacy Screening tool and classified as high (score ≥ 17) or low (< 17). Bivariate analyses were performed.

**Results:**

A total of 127 participants were recruited, with 26.8% (n = 34) having low HL. Low HL patients reported lower household incomes and lower rates of post-secondary education (11.7% vs. 45.2%, p < 0.01). Participants with low HL also reported less understanding of prescribed medications (90.6% vs. 98.8%), awareness of available treatment options (71.9% vs. 90.0%), and trust in their doctors (91.2% vs. 98.9%; all p < 0.01). Moreover, low HL patients were more likely to have challenges in making an appointment (23.5% vs. 8.6%) and miss appointments due to transportation issues (44.1% vs. 17.2%, both p < 0.01). Although there were no differences in obesity rates, low HL patients were less likely to consider bariatric surgery if indicated (61.8% vs. 81.7%) and recommend bariatric surgery to others (52.9% vs. 72.0%; both p < 0.01).

**Conclusions:**

Low HL patients in the Deep South reported less familiarity with healthcare treatment options, trust in their doctors, and likelihood to consider bariatric surgery when indicated. Community outreach and education programs addressing the unique needs of low HL patients are necessary to increase their awareness and improve their access to bariatric surgery.

**Graphical abstract:**

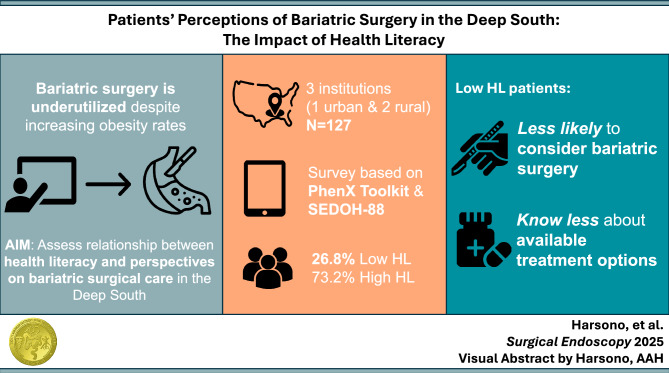

**Supplementary Information:**

The online version contains supplementary material available at 10.1007/s00464-025-11916-w.

Obesity affects more than 890 million (16%) adults globally [1] and remains an epidemic in the United States, with more than 30% of the adult population living with obesity [2]. Over time, obesity is associated with an increased risk of developing medical comorbidities, such as diabetes, heart disease, chronic kidney disease, and malignancies, as well as a higher risk of mortality [3–7]. Although multiple treatment approaches for obesity are available and accessible, many of them are generally ineffective in long-term weight control, except for more definitive strategies, such as bariatric surgery [8, 9]. Bariatric surgery has consistently been shown to have sustained effects on weight loss and even to improve patients’ quality of life [10–14].

Although bariatric surgery has demonstrated improved outcomes and safety [15, 16], the utilization of bariatric surgery for patients who meet the body mass index (BMI) criteria of the National Institute of Health (NIH) has been stagnant in the United States at 1% [17, 18] before recently reaching 5.5% [19]. However, regional variation exists in the adoption and propagation of bariatric surgery, with state-level bariatric surgery rates ranging from 2 to 10% [19]. This is particularly challenging in the Deep South region of the United States, where obesity and severe obesity rates are higher than the national average, yet bariatric surgery remains underutilized [20, 21]. Additionally, the Deep South is also notable for its unique intersection of low health literacy (HL), racial diversity, and surgical disparities [22, 23]. Previous studies showed that racial disparities exist in bariatric surgery with significant underutilization of bariatric care among Black populations [24, 25]; however, the role of HL on bariatric surgery access remains unclear and has not been thoroughly investigated.

The impact HL can have on long-term surgical outcomes has been demonstrated across surgical subspecialties, as outcomes depend not only on the operation performed, but also on education, specific health knowledge, and HL of the patients [26]. Moreover, HL is essential to weight loss and maintenance after bariatric surgery [27]. However, the impact of HL on patients’ perspectives on access to bariatric surgery is largely unknown, particularly in an area of the United States with underutilization of bariatric care. Therefore, the aim of this study was to assess the relationship between HL and patients’ perspectives on access to bariatric surgical care in the Deep South.

## Methods

### Study design, inclusion criteria, and exclusion criteria

A cross-sectional, multi-institutional study was conducted in surgery clinics at three institutions in Alabama: University of Alabama at Birmingham (UAB) Hospital in Birmingham, Whitfield Regional Medical Center in Demopolis, and Regional Medical Center of Central Alabama in Greenville. Data collection was performed from December 2023 to September 2024. Inclusion criteria were patients aged 18 years or older who were English-speaking, had undergone surgical evaluation at one of the three sites during the study period, and could provide consent. Exclusion criteria were patients who were younger than 18 years, non-English speaking, and unable to give consent. The study protocol and supplemental materials were approved by the UAB institutional review board (IRB-300010829). The Strengthening the Reporting of Observational Studies in Epidemiology (STROBE) guidelines were used for study reporting [28, 29].

### Study sites (Setting)

Two of the three healthcare facilities (Demopolis in Marengo County and Greenville in Butler County) are rural institutions located within the Black Belt of Alabama within close proximity of UAB Hospital located in Jefferson County. The Black Belt has distinct geographic, social, and cultural boundaries with high rates of poverty and health care disparities in the region [30]. According to the 2020 to 2023 US Census Bureau population estimates, Marengo, Butler, and Jefferson counties comprised 18,648, 18,382, and 674,340 residents, respectively [31]. More granularly, in 2019, the Black Belt comprised 56% Black residents, with nearly one in four individuals (23.7%) living below the poverty line [30]. These two rural healthcare facilities alongside the UAB Hospital were part of the UAB Surgery Community Network [32].

### Survey design and collection

Participants were recruited to complete a survey based on the NIH PhenX tool kit [33], assessing socioeconomic determinants of health and perception of safety, efficacy, and accessibility of bariatric surgery. The survey was based on the SEDOH-88, a comprehensive 88-item survey that captures health-related factors across multiple socioecological levels: individual (health literacy), interpersonal (physician trust), organizational (healthcare cost and insurance), and community (healthcare access and community support) [34]. Additional questions regarding patient perspectives on bariatric surgery were included in the survey based on validated instruments.

As part of an ongoing prospective study, patients undergoing surgical evaluation at each of the three sites were approached in person during preoperative encounters for informed consent and survey completion. The survey was administered by two study personnel (AAHH and NCE) in person, either verbally or filled in directly by the participants, and via email for patients who were not able to complete the survey in the clinic to maximize valid responses [35]. Answers were recorded on an iPad device linked directly into the research electronic data capture (REDCap), a secure online electronic data capture tools platform [36], which was hosted and managed by UAB REDCap servers. Each survey was completed in approximately 12–15 min.

### Exposure and outcomes

Exposures measured were HL levels as captured by the BRIEF component of the survey, a validated four-question screening tool [37–39]. The four questions involved in the survey included as follow: (1) How often do you have someone help you read the hospital materials? (2) How often do you have problems learning about your medical condition because of difficulty understanding the written information? (3) How often do you have a problem understanding what is told to you about your medical condition? and (4) How confident are you filling out medical forms by yourself? The answer for each question was rated with a 5-point Likert response scale and then summarized with a possible total range of 4 to 20. HL scores were then categorized into “High HL” (17–20) and “Low HL” (4–16) as described previously [40].

The primary outcomes of interest were the patients’ responses to the socioecological questions as well as their perceptions of access to bariatric surgery as measured by the survey. Survey answers were offered in multiple-choice-type questions, through a yes/no format, and a composite of four or five Likert scale options. Answers were mainly assessing patients’ perspectives on: 1) how agreeable the statements or questions were, which were measured with options ranging from “strongly agree”, “agree”, “neutral”, “disagree”, and “strongly disagree”; 2) how often something happens, answered by either “always”, “often”, “sometimes”, “occasionally”, and “never”; and 3) to what extend respondents feel something, answered by “not at all”, “a little”, “a moderate amount”, “very much”, and “an extreme amount”. A detailed version of the survey is attached in Supplementary 1. For analysis purposes, Likert scores “neutral”, “disagree”, and “strongly disagree” were grouped and compiled as “Negative”, whereas “strongly agree”, “agree” were grouped together as “Positive”.

### Statistical analysis

Descriptive statistics were conducted using frequencies and percentages for categorical variables, whereas continuous variables were summarized with means and SD or medians with interquartile values. Participants were stratified based on their HL level, either high or low. Bivariate analyses compared the demographics and the survey responses using the chi-square or Fisher exact probability test as appropriate for categorical variables and the Kruskal–Wallis rank-sum test for continuous variables. A p-value of < 0.05 was considered statistically significant. Data analysis was performed using SAS software version 9.4 (SAS Institute, Cary, NC).

## Results

### Demographics

Of the 140 patients approached, 127 (90.7%) participants agreed to participate and were recruited. The majority (66.1%, n = 84) of the participants were recruited from the urban hospital, whereas 32.2% (n = 41) and 11.8% (n = 15) were recruited from the two rural sites. The majority of the study participants were female (76.4%, n = 97), 99.2% were primarily English speaking, 26.8% earned below $15,000, 46.5% were employed, 57.5% were high school graduates, and 32.3% had 3 dependents in their family. A total of 73.2% (n = 93) of participants were classified as high HL, whereas 26.8% (n = 34) were classified as low HL. High HL participants were more likely to earn higher salaries of more than $15,000 (71% vs 32.3%, p < 0.001) and have higher rates of post-secondary education (45.2% vs 11.7%, p = 0.007) than low HL participants (Table 1). However, there were no significant differences in sex, primary language, number of dependents, and employment status between low and high HL participants (Table 1).

**Table 1:** Demographic characteristics of patients by health literacy level in the Deep South

Variables	Overall (N = 127)	High HL (n = 93, 73.2%)	Low HL (n = 34, 26.8%)	P-value
Institutions				0.528
UAB Hospital	80 (63.0)	61 (65.6)	19 (55.9)	
Greenville	32 (25.2)	21 (22.6)	11 (32.3)	
Demopolis	15 (11.8)	11 (11.8)	4 (11.8)	
Income category				** < 0.001**
$0—$15,000	34 (26.8%)	20 (21.5%)	14 (41.2%)	
$15,001—$30,000	22 (17.3%)	16 (17.2%)	6 (17.6%)	
$30,001—$75,000	33 (26.0%)	32 (34.4%)	1 (2.9%)	
$75,001 and above	22 (17.3%)	18 (19.4%)	4 (11.8%)	
Unknown/not stated	16 (12.6%)	7 (7.5%)	9 (26.5%)	
Gender				0.247
Female	97 (76.4%)	72 (77.4%)	25 (73.5%)	
Male	29 (22.8%)	21 (22.6%)	8 (23.5%)	
Prefer not to answer	1 (0.8%)	0 (0.0%)	1 (2.9%)	
Employment status				0.07
Employed	59 (46.5%)	48 (51.6%)	11 (32.4%)	
Disabled	32 (25.2%)	19 (20.4%)	13 (38.2%)	
Retired	18 (14.2%)	13 (14.0%)	5 (14.7%)	
Unemployed	12 (9.4%)	7 (7.5%)	5 (14.7%)	
Others	6 (4.7%)	6 (6.5%)	0 (0.0%)	
Education Level				**0.007**
Highschool graduate	73 (57.5%)	46 (49.5%)	27 (79.4%)	
College graduate or higher	27 (21.3%)	24 (25.8%)	3 (8.8%)	
Associate degree	19 (15.0%)	18 (19.4%)	1 (2.9%)	
Some schooling	8 (6.3%)	5 (5.4%)	3 (8.8%)	
Number of Dependents				0.059
1	11 (8.7%)	4 (4.3%)	7 (20.6%)	
2	24 (18.9%)	19 (20.4%)	5 (14.7%)	
3	41 (32.3%)	30 (32.3%)	11 (32.4%)	
4	20 (15.7%)	15 (16.1%)	5 (14.7%)	
5 or more	31 (24.4%)	25 (26.9%)	6 (17.6%)	

Bold font indicates statistical significance

Data are presented in n (%)

**Fig. 1:**
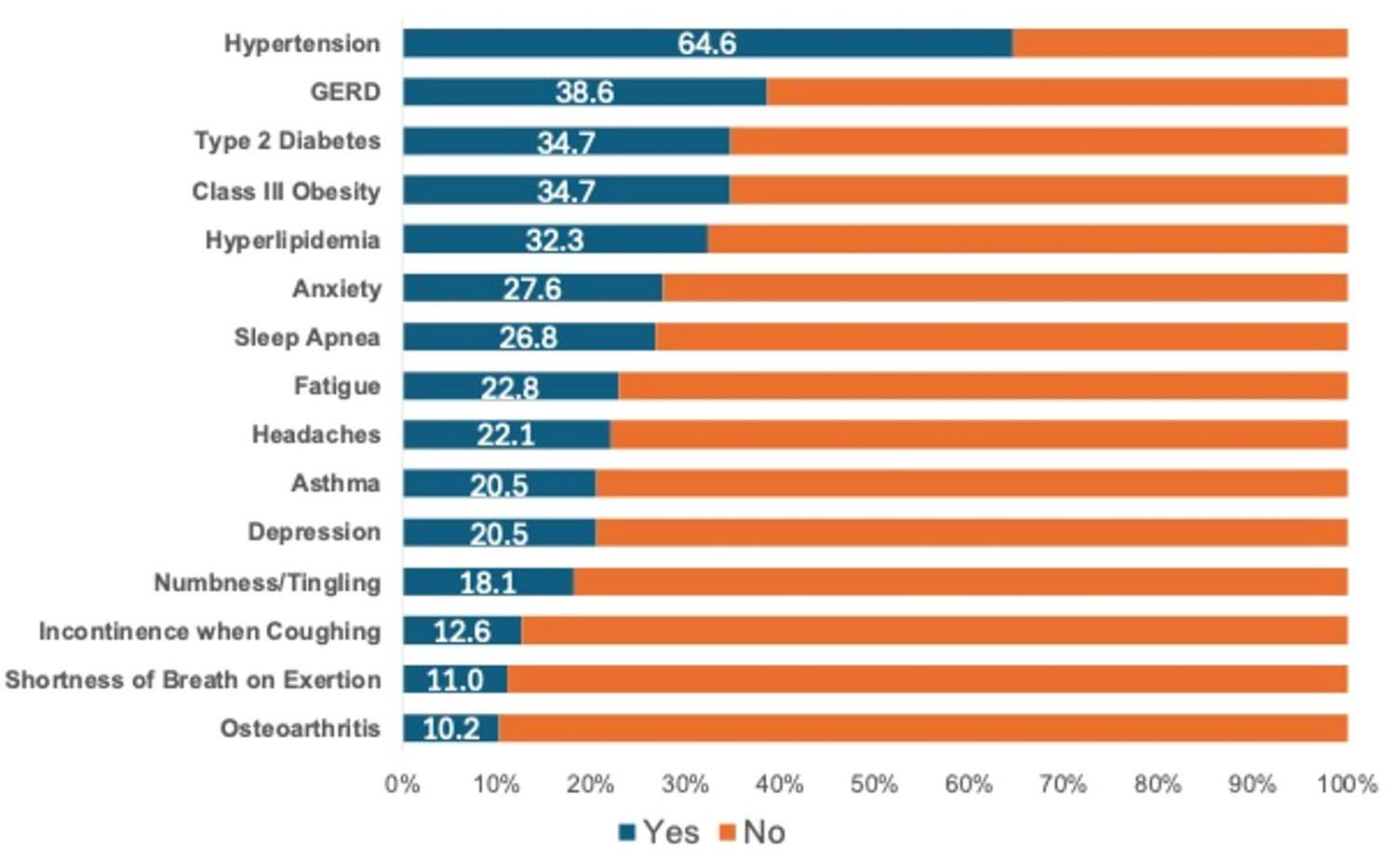
Top 15 of patients’ self-reported comorbidities

Patients’ comorbidities are shown in Fig. 1. The most common comorbidities experienced by the participants were hypertension (64.6%), followed by gastroesophageal reflux disease (38.6%), type 2 diabetes (34.7%), class III obesity (34.7%), and hyperlipidemia (32.3%). Mental health issues were also prevalent among participants, as 27.6% reported anxiety and 20.5% had history of depression. Other notable reported comorbidities were obstructive sleep apnea (26.8%), asthma (20.5%), incontinence when coughing (12.6%), shortness of breath on exertion (11%), and osteoarthritis (10.2%).

### Survey responses: patient activation measures and support

Other survey component questions were presented in Table 2. Within the patient activation measures (PAM), more than 90% of participants were aware and confident of their own role in taking care of their health (Table 2). Stratification by HL levels showed that participants with low HL reported less understanding of their prescribed medications (90.6% vs. 98.8%, p < 0.01) and were less aware of available treatment options (71.9% vs. 90.0%, p < 0.01). The majority of the participants expressed trust in doctors’ care, medical skills, believed they were careful, thought the best for the patient, and thought they were attentive to patients (Table 2). However, low HL participants were less likely to trust that their doctors’ medical skills were as they should be (91.2% vs. 98.9%, p = 0.027). Within the social support and community domain, there were no statistically significant differences between different HL levels (Table 2). In the spirituality aspect, low HL participants were less likely to feel that their life has a purpose (79.4% vs 92.5%, p = 0.037). Regarding healthcare access, low HL patients found it more challenging to make an appointment (23.5% vs. 8.6%, p = 0.025), were less likely to use internet to access social media (79.4% vs 95.7%, p = 0.004), and were more likely to miss appointments due to transportation issues (44.1% vs. 17.2%, p = 0.002).

**Table 2:** Bariatric surgery referral and bariatric surgery specific questions by health literacy levels

Questions	Overall (N = 127)	High HL (n = 93, 73.2%)	Low HL (n = 34, 26.8%)	P-value
Bariatric Surgery Referral				
If referred for bariatric surgery, I would be afraid of any surgery and avoid it unless absolutely needed				0.313
Positive	47 (37.6%)	37 (40.2%)	10 (30.3%)	
Negative	78 (62.4%)	55 (59.8%)	23 (69.7%)	
I am concerned about the risks of bariatric surgery				0.238
Positive	61 (49.6%)	48 (52.7%)	13 (40.6%)	
Negative	62 (50.4%)	43 (47.3%)	19 (59.4%)	
I am concerned about the costs associated with bariatric surgery				0.524
Positive	48 (39.0%)	34 (37.4%)	14 (43.8%)	
Negative	75 (61.0%)	57 (62.6%)	18 (56.3%)	
I am concerned about traveling to appointments and traveling for the operation				0.139
Positive	20 (16.1%)	12 (13.2%)	8 (24.2%)	
Negative	104 (83.9%)	79 (86.8%)	25 (75.8%)	
The wait time for bariatric surgery is too long				0.638
Positive	41 (33.1%)	29 (31.9%)	12 (36.4%)	
Negative	83 (66.9%)	62 (68.1%)	21 (63.6%)	
Bariatric Surgery Specific Questions				
If it were clinically indicated and you were recommended and/or referred to undergo bariatric (weight loss) surgery (i.e. sleeve gastrectomy, lap band or gastric bypass), how likely would you be to consider bariatric surgery?				**0.019**
Positive	97 (76.4%)	76 (81.7%)	21 (61.8%)	
Negative	30 (23.6%)	17 (18.3%)	13 (38.2%)	
How likely would you be to recommend bariatric surgery to others?				**0.043**
Positive	85 (66.9%)	67 (72.0%)	18 (52.9%)	
Negative	42 (33.1%)	26 (28.0%)	16 (47.1%)	
Are you obese? (Body Mass index (BMI) greater than 30)				0.166
Yes	116 (91.3%)	83 (89.2%)	33 (97.1%)	
No	11 (8.7%)	10 (10.8%)	1 (2.9%)	
Is your BMI greater than 35?				**0.007**
Yes	88 (80.7%)	68 (87.2%)	20 (64.5%)	
No	21 (19.3%)	10 (12.8%)	11 (35.5%)	
Have you been obese for greater than 5 years?				**0.007**
Yes	94 (83.2%)	73 (89.0%)	21 (67.7%)	
No	19 (16.8%)	9 (11.0%)	10 (32.3%)	
Have you been obese since childhood?				0.519
Yes	42 (37.2%)	29 (35.4%)	13 (41.9%)	
No	71 (62.8%)	53 (64.6%)	18 (58.1%)	
Have you been obese since pregnancy?				0.379
Yes	52 (46.8%)	40 (49.4%)	12 (40.0%)	
No	59 (53.2%)	41 (50.6%)	18 (60.0%)	
Have you ever been referred for bariatric surgery?				0.357
Yes	72 (56.7%)	55 (59.1%)	17 (50.0%)	
No	55 (43.3%)	38 (40.9%)	17 (50.0%)	
Have you previously had bariatric surgery?				0.051
Yes	21 (16.5%)	19 (20.4%)	2 (5.9%)	
No	106 (83.5%)	74 (79.6%)	32 (94.1%)	
Do you currently follow a special diet?				0.357
Yes	72 (56.7%)	55 (59.1%)	17 (50.0%)	
No	55 (43.3%)	38 (40.9%)	17 (50.0%)	
Have you previously dieted?				0.096
Yes	102 (80.3%)	78 (83.9%)	24 (70.6%)	
No	25 (19.7%)	15 (16.1%)	10 (29.4%)	
During the last 3 months, did you have any episodes of excessive overeating?				0.195
Yes	41 (32.3%)	27 (29.0%)	14 (41.2%)	
No	86 (67.7%)	66 (71.0%)	20 (58.8%)	

Bold font indicates statistical significance

Data presented as n (%)

A positive answer combines those who chose options “agree” and “strongly agree”, whereas a negative answer combines those who answered “neutral”, “disagree”, and “strongly disagree”

### Bariatric surgery perceptions and health literacy

Questions regarding both bariatric surgery referral and perceptions are contained in Table 3. Within the bariatric surgery referral process concerns, 37.6% (n = 47) of participants felt afraid of the bariatric surgery process when referred. A total of 49.6% (n = 61) of participants were concerned about the perioperative risks of bariatric surgery, 39% (n = 48) were concerned about the associated medical costs, 16.1% (n = 15) expressed concerns about traveling to the clinic or hospital for appointments or for their operation, whereas 33.1% (n = 41) showed concerns about waiting time for bariatric surgery evaluation. However, when stratified by HL level, there were no statistically significant differences detected between high and low HL levels with regards to perceived fear of surgery, risk, cost, traveling, and waiting time related to bariatric surgery.

**Table 3:** Survey questions on patient-activated measure (PAM), doctor’s attributes, social support, spirituality, community, and healthcare

Questions	Overall (N = 127)	High HL (n = 93, 73.2%)	Low HL (n = 34, 26.8%)	P-value
Patient Activated Measure				
I am the person responsible for taking care of my health				0.091
Positive	114 (99.1%)	85 (100.0%)	29 (96.7%)	
Negative	1 (0.9%)	0 (0.0%)	1 (3.3%)	
Taking an active role in own health care is the most important thing that affects my health				0.416
Positive	116 (98.3%)	87 (97.7%)	29 (100.0%)	
Negative	2 (1.7%)	2 (2.3%)	0 (0.0%)	
I am confident that I can help prevent or reduce problems associated with my health				0.107
Positive	115 (97.5%)	86 (98.9%)	29 (93.5%)	
Negative	3 (2.5%)	1 (1.1%)	2 (6.5%)	
I know what each of my prescribed medications do				**0.030**
Positive	113 (96.6%)	84 (98.8%)	29 (90.6%)	
Negative	4 (3.4%)	1 (1.2%)	3 (9.4%)	
I am confident that I can tell whether I need to go the doctor or whether I can take care of health problem myself				0.318
Positive	113 (94.2%)	84 (95.5%)	29 (90.6%)	
Negative	7 (5.8%)	4 (4.5%)	3 (9.4%)	
I am confident that I can tell a doctor concerns I have even when he or she does not ask				0.923
Positive	114 (96.6%)	83 (96.5%)	31 (96.9%)	
Negative	4 (3.4%)	3 (3.5%)	1 (3.1%)	
I am confident that I can follow through on medical treatments I may need to do at home				0.091
Positive	118 (99.2%)	88 (100.0%)	30 (96.8%)	
Negative	1 (0.8%)	0 (0.0%)	1 (3.2%)	
I understand my health problems and what causes them				0.060
Positive	113 (94.2%)	85 (96.6%)	28 (87.5%)	
Negative	7 (5.8%)	3 (3.4%)	4 (12.5%)	
I know what treatments are available for my health problems				**0.013**
Positive	104 (85.2%)	81 (90.0%)	23 (71.9%)	
Negative	18 (14.8%)	9 (10.0%)	9 (28.1%)	
I have been able to maintain (keep up with) lifestyle changes, like eating right or exercising				0.890
Positive	96 (82.1%)	70 (82.4%)	26 (81.3%)	
Negative	21 (17.9%)	15 (17.6%)	6 (18.8%)	
I know how to prevent the problems with my health				0.243
Positive	105 (86.8%)	80 (88.9%)	25 (80.6%)	
Negative	16 (13.2%)	10 (11.1%)	6 (19.4%)	
I am confident I can figure out solutions when new problems arise with my health				0.301
Positive	102 (85.7%)	78 (87.6%)	24 (80.0%)	
Negative	17 (14.3%)	11 (12.4%)	6 (20.0%)	
I am confident that I can maintain lifestyle changes, like eating right and exercising, even during the times of stress				0.656
Positive	104 (86.7%)	77 (87.5%)	27 (84.4%)	
Negative	16 (13.3%)	11 (12.5%)	5 (15.6%)	
Doctors' Attributes				
Sometimes your doctor cares more about what is convenient for him/her than about your medical need				0.195
Positive	15 (11.9%)	9 (9.7%)	6 (18.2%)	
Negative	111 (88.1%)	84 (90.3%)	27 (81.8%)	
Your doctor's medical skills are not as they should be?				**0.027**
Positive	4 (3.1%)	1 (1.1%)	3 (8.8%)	
Negative	123 (96.9%)	92 (98.9%)	31 (91.2%)	
Your doctor is extremely thorough and careful?				0.232
Positive	104 (82.5%)	79 (84.9%)	25 (75.8%)	
Negative	22 (17.5%)	14 (15.1%)	8 (24.2%)	
Your doctor only thinks about what is best for you				0.661
Positive	104 (81.9%)	77 (82.8%)	27 (79.4%)	
Negative	23 (18.1%)	16 (17.2%)	7 (20.6%)	
Sometimes your doctor does not pay full attention to what you are trying to tell him/her				0.288
Positive	13 (10.3%)	8 (8.6%)	5 (15.2%)	
Negative	113 (89.7%)	85 (91.4%)	28 (84.8%)	
You have no worries about putting your life in your doctor's hands				0.311
Positive	101 (79.5%)	76 (81.7%)	25 (73.5%)	
Negative	26 (20.5%)	17 (18.3%)	9 (26.5%)	
All in all, you have complete trust in your doctor				0.245
Positive	102 (80.3%)	77 (82.8%)	25 (73.5%)	
Negative	25 (19.7%)	16 (17.2%)	9 (26.5%)	
Social Support				
If you needed it how often is someone available to take you to the doctor?				0.341
Positive	98 (78.4%)	71 (76.3%)	27 (84.4%)	
Negative	27 (21.6%)	22 (23.7%)	5 (15.6%)	
Who understands your problems?				0.276
Positive	94 (74.6%)	71 (77.2%)	23 (67.6%)	
Negative	32 (25.4%)	21 (22.8%)	11 (32.4%)	
To love and make you feel wanted?				0.351
Positive	103 (81.7%)	77 (83.7%)	26 (76.5%)	
Negative	23 (18.3%)	15 (16.3%)	8 (23.5%)	
To help you if you were confined to bed?				0.430
Positive	105 (84.0%)	75 (82.4%)	30 (88.2%)	
Negative	20 (16.0%)	16 (17.6%)	4 (11.8%)	
To help with daily chores if you were sick?				0.946
Positive	96 (76.2%)	71 (76.3%)	25 (75.8%)	
Negative	30 (23.8%)	22 (23.7%)	8 (24.2%)	
To prepare your meals if you are unable to do it yourself?				0.727
Positive	99 (78.6%)	73 (79.3%)	26 (76.5%)	
Negative	27 (21.4%)	19 (20.7%)	8 (23.5%)	
To have a good time with?				0.102
Positive	101 (80.2%)	77 (83.7%)	24 (70.6%)	
Negative	25 (19.8%)	15 (16.3%)	10 (29.4%)	
To turn to for suggestions about how to deal with a personal problem?				0.575
Positive	97 (77.0%)	72 (78.3%)	25 (73.5%)	
Negative	29 (23.0%)	20 (21.7%)	9 (26.5%)	
Spirituality				
To what extent do you feel your life has a purpose?				**0.037**
Positive	113 (88.9%)	86 (92.5%)	27 (79.4%)	
Negative	14 (11.1%)	7 (7.5%)	7 (20.6%)	
To what extent does faith contribute to your wellbeing?				0.681
Positive	110 (87.3%)	81 (88.0%)	29 (85.3%)	
Negative	16 (12.7%)	11 (12.0%)	5 (14.7%)	
Community				
In the last 12 months, were you ever hungry but didn't eat because you couldn't afford enough food?				0.562
Yes	19 (15.1%)	13 (14.0%)	6 (18.2%)	
No	107 (84.9%)	80 (86.0%)	27 (81.8%)	
At any time in your life, have you ever been unfairly fired?				0.992
Yes	41 (32.3%)	30 (32.3%)	11 (32.4%)	
No	86 (67.7%)	63 (67.7%)	23 (67.6%)	
For unfair reasons, have you ever not been hired for a job?				0.748
Yes	42 (33.1%)	30 (32.3%)	12 (35.3%)	
No	85 (66.9%)	63 (67.7%)	22 (64.7%)	
Have you ever been unfairly denied a promotion?				0.322
Yes	33 (26.0%)	22 (23.7%)	11 (32.4%)	
No	94 (74.0%)	71 (76.3%)	23 (67.6%)	
Have you ever been unfairly stopped, searched, questioned, physically threatened or abused by the police?				0.264
Yes	22 (17.3%)	14 (15.1%)	8 (23.5%)	
No	105 (82.7%)	79 (84.9%)	26 (76.5%)	
Have you ever moved into a neighborhood where neighbors made life difficult for you or your family?				0.753
Yes	24 (19.4%)	17 (18.7%)	7 (21.2%)	
No	100 (80.6%)	74 (81.3%)	26 (78.8%)	
Have you ever been unfairly discouraged by a teacher or advisor from continuing your education?				0.415
Yes	21 (16.7%)	17 (18.3%)	4 (12.1%)	
No	105 (83.3%)	76 (81.7%)	29 (87.9%)	
Have you ever received service from someone such as a plumber or car mechanic that was worse than what other people get?				0.045
Yes	33 (26.2%)	20 (21.5%)	13 (39.4%)	
No	93 (73.8%)	73 (78.5%)	20 (60.6%)	
Health Care				
How easy is it for you to make an appointment if you are sick and need health care?				**0.025**
Easy	111 (87.4%)	85 (91.4%)	26 (76.5%)	
Difficult	16 (12.6%)	8 (8.6%)	8 (23.5%)	
During the past 12 months, have you delayed or not gotten medical care because of the cost?				0.557
Yes	22 (17.3%)	15 (16.1%)	7 (20.6%)	
No	105 (82.7%)	78 (83.9%)	27 (79.4%)	
Has your tablet /smartphone helped you with your health goals?				0.122
Yes	98 (77.2%)	75 (80.6%)	23 (67.6%)	
No	29 (22.8%)	18 (19.4%)	11 (32.4%)	
In the past 12 months, have you used the internet to access any social media?				**0.004**
Yes	116 (91.3%)	89 (95.7%)	27 (79.4%)	
No	11 (8.7%)	4 (4.3%)	7 (20.6%)	
Are you currently covered by any of the insurance plans?				0.097
Yes	126 (99.2%)	93 (100.0%)	33 (97.1%)	
No	1 (0.8%)	0 (0.0%)	1 (2.9%)	
Have you ever missed a doctor's appointment because of transportation problems?				**0.002**
Yes	31 (24.4%)	16 (17.2%)	15 (44.1%)	
No	96 (75.6%)	77 (82.8%)	19 (55.9%)	

Bold font indicates statistical significance

Data presented as n (%)

A positive answer combines those who chose options “agree” and “strongly agree”, whereas a negative answer combines those who answered “neutral”, “disagree”, and “strongly disagree”

**Fig. 2:**
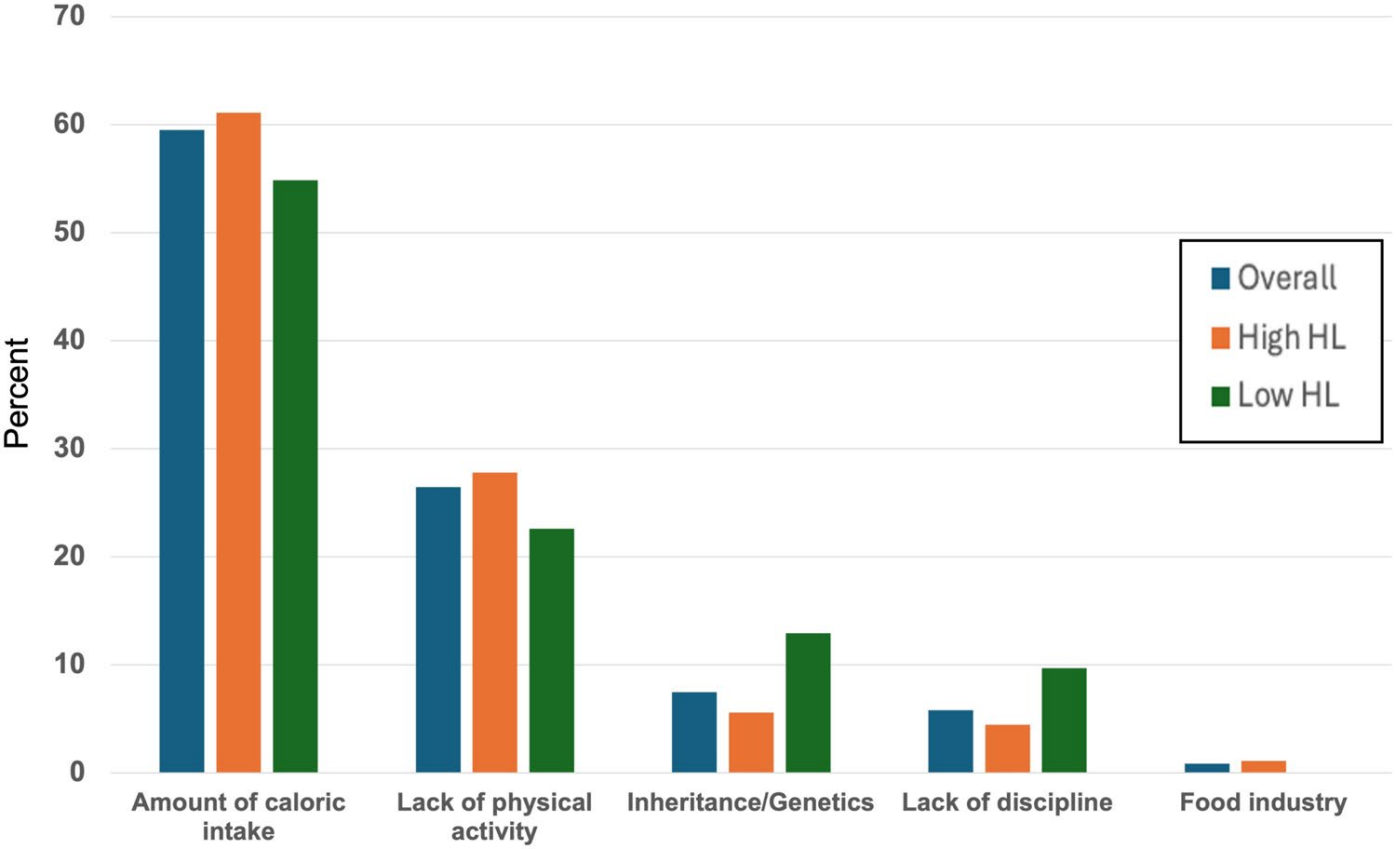
Patients’ perceptions on what is mainly responsible for obesity by health literacy levels

A total of 76.4% (n = 97) of participants reported they would consider having bariatric surgery if it were clinically indicated, and this number was consistently higher among participants with high HL (81.7% vs 61.8%, p = 0.019). The majority of participants (66.9%, n = 85) also reported that they would recommend bariatric surgery to others, which was more commonly reported among participants with high HL compared with low HL participants (72% vs 52.9%, p = 0.043). When assessing bariatric surgery history, 16.5% patients (n = 21) had undergone bariatric surgery previously. Patients with higher HL were also more aware of having a BMI greater than 35 kg/m^2^ (87.2% vs 64.5%, p = 0.007), and had obesity for more than 5 years (89% vs 67.7%, p = 0.007). As shown in Table 3, there was no significant difference between HL levels in participants’ history of having obesity since childhood or pregnancy. Overall, 56.7% (n = 72) had been referred for bariatric surgery. Approximately 80% (n = 102) of participants had previously dieted, while 56.7% (n = 72) of the participants reported currently following a special diet, and 32.3% (n = 41) had experienced episodes of overeating. There were no statistically significant differences for dieting between different HL levels. When asked about the leading cause for obesity (Fig. 2), more than half of participants thought amount of caloric intake, followed by lack of physical activity, inheritance/genetics, lack of discipline, and lastly food industry, and this was consistent among HL levels (Table 3).

## Discussion

This multi-institutional study assessed the association between HL and patients’ access to healthcare and perceptions of bariatric surgical care in a diverse patient population in the Deep South. Participants with low HL found it much more challenging to navigate healthcare systems, with higher reports of difficulties with transportation to appointments, difficulties making appointments, and lower reported trust in their physicians. Additionally, patients with low HL were less likely to consider having bariatric surgery and to recommend it to others. Survey respondents expressed some concerns about bariatric surgery, especially the perioperative risks, associated costs, and the waiting time for surgery, but there were no differences in these reported concerns on the basis of HL.

Although significant disparities in bariatric surgery access have been associated with minority race, lower income, sex, and insurance types, other factors such as HL may play a role in access to bariatric care [24]. Not surprisingly, the findings from this multi-institutional survey demonstrated that lower HL was more prevalent among participants with lower income and educational levels. Similarly, Altiner et al. also found that patients with lower income and educational levels had significantly lower HL levels [41]. Additionally, the survey responses from this study also indicated that HL levels were also associated with patients’ perceptions of bariatric surgery and their willingness to undergo bariatric surgery. These findings are consistent with prior work by Hecht et al*.* which demonstrated that having adequate HL was a significant predictor for undergoing bariatric surgery [42]. There are several possible explanations for why patients with lower HL are less likely to undergo bariatric surgery. Consistent with the findings of this study, prior work has also indicated that patients with lower HL have a higher risk of missing appointments [27, 43–45], which can also increase their likelihood of loss to follow-up preoperatively [42]. Likewise, patients with lower HL may not communicate with providers when/if they do not understand information and may even ask fewer clarifying questions to providers, although they may have difficulty digesting the information [46, 47]. Thus, with a lower quality of communication and comprehension, patients with low HL may not follow through with the initial plan of having surgery. Moreover, prior work has indicated that among those who do achieve bariatric surgery, higher HL was associated with superior postoperative weight loss [48] and weight maintenance [43]. Consequently, the vital role that HL can play along the entire continuum of bariatric surgical care, from access to surgery through the maintenance period, cannot be emphasized enough.

This study confirmed that some of the barriers to bariatric surgery remain concerns regarding operative risks, waiting time, and cost. Bariatric surgery has evolved within the last several decades, with significant improvement in surgical outcomes and risk minimization [13, 49, 50]. Thus, there is opportunity to improve engagement with patients in a shared decision-making conversation about how the risks and benefits of surgery have progressed, with superior durable outcomes compared with medical and lifestyle management alone. Previous studies have shown that patients with Medicaid insurance experienced longer wait times from initial entry into a bariatric surgery program to operation, compared with other patients [51]. Moreover, although the cost of bariatric surgery seems expensive, compared with other treatments, bariatric surgery is a cost-saving option over a lifetime [52, 53]. Furthermore, as patients with low HL reported less trust in their physicians and less reported interest in bariatric surgery, an opportunity exists for community engagement in underserved areas with higher rates of low HL.

Future steps of the study would be the implementation of targetable interventions involving community outreach and engagement to allow for the establishment of better communication, patient education, and improved patient relationships in communities with some of the highest needs for surgical and bariatric care. From the healthcare providers' side, providers’ education to improve awareness of low HL patients, as well as applying HL-sensitive tools, such as the teach-back method, would bridge the gap and address the HL disparities [54]. From the patient side, the utilization of HL-sensitive educational videos targeting the low HL population had also been proven to improve patients’ HL levels [55]. Moreover, further steps could also involve the utilization of telehealth to address the barriers and improve access to bariatric surgery, alongside addressing the HL disparities as shown in our study [54, 56, 57].

There are several limitations in our study. First, we cannot establish causality between the identified SDOH components within the survey and HL due to the nature of the cross-sectional study. As the study captures a snapshot of participants’ single moment in time, this finding may not reflect the dynamic changes in behavior and exposure patterns, such as HL levels and their perceptions. Although a survey-type study is subject to non-response bias, more than 95% of the survey questions were answered by 90.7% of our participants. This finding fulfills the 80% minimum completion rate requirement to decrease such bias as recommended by the American Association for Public Opinion Research [35, 58, 59]. Second, there is still a potential of measurement and recall bias of survey-type study as it is self-reported by nature. Thus, we have adapted a previously validated survey [34] with objective measures of which components are covered within the NIH PhenX toolkit [33] as a strategy to reduce such biases. Third, the study may be underpowered to detect several meaningful associations of the SDOH components covered within our survey. The sample size limits our ability to perform multivariable analysis to control for factors known to influence perceptions of bariatric surgery, such as age, sex, and race. Unfortunately, as such analyses were not performed, we could not comment on which SDOH has the highest association with bariatric surgery perception. However, our findings highlighted novel associations of bariatric surgery perceptions among patients in the Deep South, which have been historically understudied. Furthermore, our study lacked the utilization of control groups or comparison cohorts outside the Deep South. Despite the specificity of the sample location, patients in the Deep South had unique characteristics, such as high proportion of underserved population. Additionally, although previous studies showed HL level gaps between rural and urban areas [60, 61], patients with low HL were also found in urban areas and areas with more resources [62]. Therefore, future studies could utilize the same tool and adopt a comparison cohort to identify regional variabilities and provide more widely representative findings. Finally, since our cohort only involved English-speaking patients from Alabama, the results may not be generalizable to non-English speaking patients, especially those residing in other regions of the country with different sociodemographic characteristics.

## Conclusion

Based on this multicenter study in the Deep South, patients with low HL reported less familiarity with healthcare treatment options and less trust in their doctors compared with their high HL counterparts. Patients with low HL were also less likely to consider bariatric surgery if it were indicated. Further qualitative work aimed at identifying barriers to access for bariatric surgery among patients with low HL are needed in order to capture more modifiable targets for intervention. Moreover, community outreach and education programs addressing the unique needs of low HL patients are necessary in order to increase their awareness and improve their access to bariatric surgery.

## Supplementary Information

Below is the link to the electronic supplementary material.

Supplementary file1 (PDF 89 KB)
