# Supplementary material for: Patients’ perceptions of bariatric surgery in the Deep South: the impact of health literacy
Source: Surg Endosc. 2025 Jul 8;39(9):6018–31. doi: 10.1007/s00464-025-11916-w (PMC12408725; doi:10.1007/s00464-025-11916-w)
Supplement: Supplementary file 1 — Supplementary file1 (PDF 89 KB) [file 464_2025_11916_MOESM1_ESM.pdf]

BARIATRIC SURGERY SURVEYS

PATIENT FULL NAME:

TODAY'S DATE-

Instructions: Please complete the surveys to the best of your ability.
Please mark X or a check to indicate your response

<u>Section A- General Information-</u>

1. **Which of the following categories best describes your total combined household income for the past 12 months?**
    a) 0 - $5,000
    b) $5,001 - $10,000
    c) $10,001 - $15,000
    d) $15,001 - $20,000
    e) $20,001 - $25,000
    f) $25,001 - $30,000
    g) $30,001 - $35,000
    h) $35,001 - $40,000
    i) $40,001 - $50,000
    j) $50,001 - $75,000
    k) $75,001 - $100,000
    l) $100,001 - $150,000
    m) $150,000 +
    n) Don't know
    o) Would rather not say

1b**. How many people (kids and adults) are currently dependent on this income and living in your household, including yourself? (drop down)**

(Answer is # of people in household dependent on income)

1c. **Of these people, how many are 0-17 years old? (drop down)**

(Answer is # of people in household 0-17 years old)

2. **What terms best express how you describe your gender identity? (Check all that apply)**

[ ] Man **[ ]** Woman **[ ]** Non-binary **[ ]** Transgender **[ ]** None of these describe me **[ ]** Prefer not to answer

3. **Which of the following best represents how you think of yourself?**

[ ] Straight; that is, not gay or lesbian, etc.
[ ] Gay
[ ] Lesbian
[ ] Bisexual
[ ] None of these describe me, and I'd like to see additional options
Branching logic: If 'none of these describe me, and I'd like to see additional options' selected:

3a. Are any of these a closer description of how you think of yourself?

[ ] Queer **[ ]** Polysexual, omnisexual, sapiosexual or pansexual **[ ]** Asexual **[ ]** Two-spirit
[ ] Have not figured out or are in the process of figuring out your sexuality
[ ] Mostly straight, but sometimes attracted to people of your own sex
[ ] Do not think of yourself as having sexuality
[ ] Do not use labels to identity yourself
[ ] Don't know the answer **[ ]** No, I mean something else (optional free text) ___________________

[ ] Prefer not to answer

4. **What is the primary language you speak at home?**
   [ ] English **[ ]** Other

4a.**Since you speak a language other than English at home, we are interested in your own opinion of how well you speak English. Would you say you speak English?**
[ ] Very well **[ ]** Not at all **[ ]** Well **[ ]** Refuse to answer
[ ] Not well **[ ]** Don't know

Section B- Education and Employment.

5. **We would like to know about what you do-are you working now, looking for work, retired, keeping house, a student, or what?**

[ ] Employed **[ ]** Only Temporarily laid off, on sick or maternity leave
[ ] Unemployed
[ ] Retire
[ ] Student
[ ] Disabled
[ ] Keeping house **[ ]** OTHER (SPECIFY):

6. **What is the highest grade or level of school you have completed or the highest degree you have received?**

[ ] NEVER ATTENDED **[ ]** Some Schooling
[ ] High school Graduate **[ ]** GED or equivalent
[ ] Some College, No Degree.
[ ] Associate degree- Non Academic
[ ] Associate degree- Academic
[ ] Bachelor's degree **[ ]** Master's degree
[ ] Professional School degree **[ ]** Doctoral degree
[ ] Refuse to answer **[ ]**Don't know

Section C-Health Care: (relates to the hospital interaction)

Access to Healthcare:

7. **About how long has it been since you last saw a doctor or other health care professional about your health?**
   [ ] Never **[ ]** Within the past year
   [ ] Within the last 2 years **[ ]** Within the last 3 years
   [ ] 10 years ago or more **[ ]** Refuse to answer
   [ ] Don't know

8. **Is there a place that you USUALLY go to if you are sick and need health care?**
   [ ] Yes **[ ]** There is NO place
   [ ] There is more than one place **[ ]** Refuse to answer
   [ ] Don't know

9. **What kind of place is it/do you go to most often?**
   [ ] A doctor's office [ ] Walk-in clinic, urgent care center
   [ ] Emergency room [ ] A VA clinic [ ] Does not go to one place often
   [ ] Refuse to answer [ ] Dont know

10. **How easy is it for you to make an appointment if you are sick and need health care?**
[ ] Very Easy [ ] Easy [ ] Neither easy nor difficult [ ] Difficult [ ] Very difficult

11. **During the past 12 months, have you delayed or not gotten medical care because of the cost?**
[ ] Yes [ ] No

12. **Has your tablet /smartphone helped you with your health goals?**
[ ] Yes [ ] No

13. **In the past 12 months, have you used the internet to access any social media?**
[ ] Yes [ ] No

14. **Are you currently covered by any of the insurance plans?**
[ ] Yes [ ] No

15. **If selected no coverage- ask- does this mean that you currently have no health coverage plan?**
[ ] Yes [ ] No

16. **Have you ever missed a doctor's appointment because of transportation problems?**
[ ] Yes [ ] No

Health Literacy

17. **How often do you have someone help you read the hospital materials?**
    [ ] Always [ ] Often [ ] Sometimes [ ] Occasionally [ ] Never

18. **How often do you have problems learning about your medical condition because of difficulty understanding the written information?**
    [ ] Always [ ] Often [ ] Sometimes [ ] Occasionally [ ] Never

19. **How often do you have a problem understanding what is told to you about your medical condition?**
    [ ] Always [ ] Often [ ] Sometimes [ ] Occasionally [ ] Never

20. **How confident are you filling out medical forms by yourself?**
    [ ] Always [ ] Often [ ] Sometimes [ ] Occasionally [ ] Never

Patient Activated Measure:
Below are the statements people make when they talk about their health. Please circle the answer that is most true for you today. If a statement does not apply, select N/A.

21. **I am the person responsible for taking care of my health.**
    [ ] Strongly disagree [ ] Disagree [ ] Agree [ ] Strongly Agree [ ] N/A

22. **Taking an active role in own health care is the most important thing that affects my health.**
[ ] Strongly disagree [ ] Disagree [ ] Agree [ ] Strongly Agree [ ] N/A

23. **I am confident that I can help prevent or reduce problems associated with my health.**
[ ] Strongly disagree [ ] Disagree [ ] Agree [ ] Strongly Agree [ ] N/A

24. **I know what each of my prescribed medications do.**
[ ] Strongly disagree [ ] Disagree [ ] Agree [ ] Strongly Agree [ ] N/A

25. **I am confident that I can tell whether I need to go the doctor or whether I can take care of health problem myself.**
[ ] Strongly disagree [ ] Disagree [ ] Agree [ ] Strongly Agree [ ] N/A

26. **I am confident that I can tell a doctor concerns I have even when he or she does not ask.**
[ ] Strongly disagree [ ] Disagree [ ] Agree [ ] Strongly Agree [ ] N/A

27. **I am confident that I can follow through on medical treatments I may need to do at home**.
[ ] Strongly disagree [ ] Disagree [ ] Agree [ ] Strongly Agree [ ] N/A

28. **I understand my health problems and what causes them.**
[ ] Strongly disagree [ ] Disagree [ ] Agree [ ] Strongly Agree [ ] N/A

29. **I know what treatments are available for my health problems.**
[ ] Strongly disagree [ ] Disagree [ ] Agree [ ] Strongly Agree [ ] N/A

30. **I have been able to maintain (keep up with) lifestyle changes, like eating right or exercising**.
[ ] Strongly disagree [ ] Disagree [ ] Agree [ ] Strongly Agree [ ] N/A

31. **I know how to prevent the problems with my health.**
[ ] Strongly disagree [ ] Disagree [ ] Agree [ ] Strongly Agree [ ] N/A

32. **I am confident I can figure out solutions when new problems arise with my health.**
[ ] Strongly disagree [ ] Disagree [ ] Agree [ ] Strongly Agree [ ] N/A

33. **I am confident that I can maintain lifestyle changes, like eating right and exercising, even during the times of stress**.
[ ] Strongly disagree [ ] Disagree [ ] Agree [ ] Strongly Agree [ ] N/A

Your Doctor:

34. **Sometimes your doctor cares more about what is convenient for him/her than about your medical need.**
[ ] Strongly Agree [ ] Agree [ ] Neutral [ ] Disagree [ ] Strongly disagree

35. **Your doctor's medical skills are not as they should be?**
[ ] Strongly Agree [ ] Agree [ ] Neutral [ ] Disagree [ ] Strongly disagree

36. **Your doctor is extremely thorough and careful**?
[ ] Strongly Agree [ ] Agree [ ] Neutral [ ] Disagree [ ] Strongly disagree

37. **Your doctor only thinks about what is best for you.**
[ ] Strongly Agree [ ] Agree [ ] Neutral [ ] Disagree [ ] Strongly disagree

38. **Sometimes your doctor does not pay full attention to what you are trying to tell him/her.**
[ ] Strongly Agree [ ] Agree [ ] Neutral [ ] Disagree [ ] Strongly disagree

39. **You have no worries about putting your life in your doctor's hands.**
[ ] Strongly Agree [ ] Agree [ ] Neutral [ ] Disagree [ ] Strongly disagree

40. **All in all, you have complete trust in your doctor.**
[ ] Strongly Agree [ ] Agree [ ] Neutral [ ] Disagree [ ] Strongly disagree

Section D - Environment Questions:

Social Support

People sometimes look to others for companionship, assistance, or other types of support. How often is each of the following kinds of support available to you if you need it? Choose one number from each line.

41. **If you needed it how often is someone available to take you to the doctor?**
[ ] None of the time [ ] A little of the time
[ ] Some of the time [ ] Most of the time
[ ] All of the time

42. **Who understands your problems?**
[ ] None of the time [ ] A little of the time
[ ] Some of the time [ ] Most of the time
[ ] All of the time

43. **To love and make you feel wanted?**
[ ] None of the time [ ] A little of the time
[ ] Some of the time [ ] Most of the time
[ ] All of the time

44. **To help you if you were confined to bed?**
[ ] None of the time [ ] A little of the time
[ ] Some of the time [ ] Most of the time
[ ] All of the time

45. **To help with daily chores if you were sick?**
[ ] None of the time [ ] A little of the time
[ ] Some of the time [ ] Most of the time
[ ] All of the time

46. **to prepare your meals if you are unable to do it yourself?**
[ ] None of the time [ ] A little of the time
[ ] Some of the time [ ] Most of the time
[ ] All of the time

47. **to have a good time with?**
[ ] None of the time [ ] A little of the time
[ ] Some of the time [ ] Most of the time
[ ] All of the time

48. **to turn to for suggestions about how to deal with a personal problem?**

[ ] None of the time **[ ]** A little of the time
[ ] Some of the time **[ ]** Most of the time
[ ] All of the time

Spirituality

49. **To what extent do you feel your life has a purpose?**

[ ] Not at all [ ] A little [ ] A moderate amount [ ] Very much [ ] An extreme amount

50. **To what extent does faith contribute to your wellbeing?**

[ ] Not at all [ ] A little [ ] A moderate amount [ ] Very much [ ] An extreme amount

Community

51. **In the last 12 months, were you ever hungry but didn't eat because you couldn't afford enough food?**

[ ] Yes **[ ]** No **[ ]** Refuse to answer **[ ]** Don't know

52. **At any time in your life, have you ever been unfairly fired?**

[ ] Yes **[ ]** No

53. **For unfair reasons, have you ever not been hired for a job**

[ ] Yes **[ ]** No

54. **Have you ever been unfairly denied a promotion?**

[ ] Yes **[ ]** No

55. **Have you ever been unfairly stopped, searched, questioned, physically threatened or abused by the police?**

[ ] Yes **[ ]** No

56. **Have you ever moved into a neighborhood where neighbors made life difficult for you or your family?**

[ ] Yes **[ ]** No

57. **Have you ever been unfairly discouraged by a teacher or advisor from continuing your education?**

[ ] Yes **[ ]** No

58. **Have you ever received service from someone such as a plumber or car mechanic that was worse than what other people get?**

[ ] Yes **[ ]** No

<u>BARIATRIC SURGERY SPECIFIC QUESTIONS</u>:

59. If it were clinically indicated and you were recommended and/or referred to undergo bariatric (weight loss) surgery (ie sleeve gastrectomy, lap band or gastric bypass), how likely would you be to consider bariatric surgery?
    a) Strongly disagree

b) Disagree
c) Neutral
d) Agree
e) Strongly Agree

60. How likely would you be to recommend bariatric surgery to others?
a) Strongly disagree
b) Disagree
c) Neutral
d) Agree
e) Strongly Agree

61. Have you ever been referred for bariatric surgery?
a) Yes
b) No

62. Have you previously had bariatric surgery?
a) Yes
b) No

Check any surgery that applies to you.
¨ Sleeve Gastrectomy
¨ Gastric Bypass
¨ Lap Band
¨ Duodenal Switch
¨ Other Bariatric Surgery (List here:_______________________________________)

62. Do you currently follow a special diet?
a) Yes
b) No

63. Have you previously dieted?
a) Yes
b) No

64. During the last 3 months, did you have any episodes of excessive overeating?
a) Yes
b) No

65. <u>Medical History</u>
Check any condition that applies to you
¨ Problems with Anesthesia ¨ Fatigue ¨ Numbness/Tingling
¨ Acid Reflux ¨ Fibromyalgia ¨ Osteoarthritis
¨ Angina ¨ Gallbladder Disorder ¨ Peripheral Vascular Disease
¨ Anxiety ¨ GI Ulcer ¨ Polycystic Ovarian Syndrome
¨ Asthma ¨ Gout ¨ Pseudotumor Cerebri

¨ Bipolar Disorder ¨ Headaches ¨ Rash/Skin Problem
¨ Bleeding ¨ Heart Attack ¨ Rheumatoid Arthritis
¨ Blood Clot ¨ Hearing Loss ¨ Scleroderma
¨ Blood Transfusion ¨ High Blood Pressure ¨ Seizures
¨ Cardiomyopathy ¨ High Cholesterol ¨ Shortness of Breath/ on Exertion
¨ Carpal Tunnel ¨ Hyperthyroidism ¨ Sleep Apnea
¨ Cirrhosis ¨ Hypothyroidism ¨ Snoring
¨ Clotting Disorder ¨ Insomnia ¨ Leak Urine when Cough/Sneeze
¨ Congestive Heart Failure ¨ Irritable Bowel Syndrome ¨ Stroke
¨ COPD ¨ Liver Disease ¨ Supraventricular Tachycardia
¨ Depression ¨ Low Blood Sugar ¨ Swelling in Legs
¨ Diabetes Type 1 ¨ Lupus ¨ Varicose Veins
¨ Diabetes Type 2 ¨ Malignant Hyperthermia ¨ Vision Problems
¨ Difficulty Swallowing ¨ Metabolic Disorder ¨ Other ______________________
¨ Dizzy/Loss of Balance ¨ Morbid Obesity

66. Obesity History
Are you obese? (Body Mass index (BMI) greater than 30) “yes” No
Is your BMI greater than 35? ¨ Yes ¨ No

If you answered yes to one of the above:
Have you been obese for greater than 5 years? ¨ Yes ¨ No
Have you been obese since childhood? Yes ¨ No
Have you been obese since pregnancy? ¨ Yes ¨ No

67. Where have you heard/read about bariatric surgery in the past? (please select all that apply)
a) Primary Care Physician
b) Weight loss medicine clinic
c) Bariatric surgeon
d) Family member/spouse
e) Friend/colleague
f) Social media
g) Websites
h) Other, please list: ____________________________

Please answer the following questions about referral for bariatric surgery (if it were clinically indicated).

68. If referred for bariatric surgery, I would be afraid of any surgery and avoid it unless absolutely needed.
[ ] Strongly Agree **[ ]** Agree **[ ]** Neutral **[ ]** Disagree **[ ]** Strongly disagree

69. I am concerned about the risks of bariatric surgery
[ ] Strongly Agree **[ ]** Agree **[ ]** Neutral **[ ]** Disagree **[ ]** Strongly disagree

70. I am concerned about the costs associated with bariatric surgery.

[ ] Strongly Agree **[ ]** Agree **[ ]** Neutral **[ ]** Disagree **[ ]** Strongly disagree

71. I am concerned about traveling to appointments and traveling for the operation.
[ ] Strongly Agree **[ ]** Agree **[ ]** Neutral **[ ]** Disagree **[ ]** Strongly disagree

72. The wait time for bariatric surgery is too long.
[ ] Strongly Agree **[ ]** Agree **[ ]** Neutral **[ ]** Disagree **[ ]** Strongly disagree

73. In your opinion: what is mainly responsible for obesity? (please choose at least one or at most 3 factors)
    a. Amount of caloric intake
    b. Lack of physical activity
    c. Inheritance/Genetics
    d. Lack of discipline
    e. Psychological trauma
    f. Social environment (e.g. family eating behavior)
    g. Food industry
    h. Lack of knowledge
    i. Other (Please list)

74. Is bariatric surgery a reasonable option for weight loss? Why or why not?

Now that you have completed the survey, we would like to ask you some questions about your experience today.

1. Thinking about the number of questions on this survey, did you feel that the survey was:
    a. Unacceptable
    b. Very burdensome
    c. Somewhat burdensome
    d. A little burdensome
    e. Not at all burdensome
2. Do you think it is appropriate to be asked about your social and economic needs at this clinic?
    b) Very inappropriate
    c) Somewhat inappropriate
    d) Neither appropriate nor inappropriate
    e) Somewhat appropriate
    f) Very appropriate
3. How much do you agree with the following statement?
I feel that my healthcare team is able to provide better care for me when they know this information about me.

    a. Strongly disagree
    b. Disagree
    c. Neutral
    d. Agree
    e. Strongly Agree
4. How does it make you feel that your healthcare team wants to collect this information?
    a. Angry
    b. Nervous
    c. Annoyed
    d. Neutral
    e. Surprised
    f. Happy
    g. Hopeful
    h. Other:___________
5. How much do you agree with the following statement?
   I feel that providing this information to my healthcare team was worth my time.
    a. Strongly disagree
    b. Disagree
    c. Neutral
    d. Agree
    e. Strongly Agree
6. How much do you agree with the following statement?
   The questions in this survey were asked in a way that I could understand.
    a. Strongly disagree
    b. Disagree
    c. Neutral
    d. Agree
    e. Strongly Agree
7. How much do you agree with the following statement?
   This survey captured details about my life that are important for my health.
    a. Strongly disagree
    b. Disagree
    c. Neutral
    d. Agree
    e. Strongly Agree
8. Which of the following do you believe have an impact on your surgery: (check all that apply)
    a. Transportation
    b. Social support
    c. Food insecurity
    d. General demographics, education, and income
    e. Employment
    f. Religious faith and purpose in life
    g. Discrimination
    h. How well you maintain your health and understand your health conditions
    i. Ability to understand hospital materials
    j. How much I trust my physician
    k. How easy it is for me to access healthcare
    l. Gender identity and sexuality

   m. None of these
   n. Other:________

9. Thinking about taking this survey on the iPad, how easy was it for you to complete?
   a. Extremely easy
   b. Somewhat easy
   c. Neutral
   d. Somewhat difficult
   e. Extremely difficult
10. How much do you agree with the following statement?
    I had enough time to complete the full length of the survey during today's visit.
    a. Strongly agree
    b. Agree
    c. Neutral
    d. Disagree
    e. Strongly disagree
11. How much do you agree with the following statement?
    I feel that this survey was smoothly integrated into today's visit.
    a. Strongly disagree
    b. Disagree
    c. Neutral
    d. Agree
    e. Strongly Agree
12. How much do you agree with the following statement?
    All in all, I feel that most patients would have no trouble completing this survey.
    a. Strongly disagree
    b. Disagree
    c. Neutral
    d. Agree
    e. Strongly Agree
